# Movable Kidney

**Published:** 1904-01-09

**Authors:** 


### MOVABLE KIDNEY.

THERE is no doubt a tendency to make too much of the importance of a movable kidney as a factor in the causation of troublesome and possibly disabling symptoms. It has been shown by more than one writer that the condition is very common indeed and is to be found if a search be made for it in a large percentage of all female patients. Moreover, in a considerable number of instances where certain symptoms have been attributed to this condition and a successful nephrorraphy has been performed, the patient has experienced no benefit whatever from the operation.

In order to ascertain the comparative frequency of movable kidney, Dr. R. C. Larrabee[1] examined 272 patients in the female medical out-patient department of the Boston City Hospital. The cases were not selected but taken at random without reference to the symptoms for which the patients attended. Each patient was examined bimanually in the dorsal position, and any tumour of the proper size, shape and consistency which could be pushed back into the normal position of the kidney was accepted as a movable kidney. Out of the total number examined one or both kidneys could be felt in 112, or $41\frac{1}{2}$ per cent. It is likely that even this figure is too low, because some of the patients were so obese that satisfactory palpation was impossible, and these cases have been classed among those in whom movable kidney was not found. The right kidney alone was affected in 98 of the 112 cases. Both kidneys were palpable in 13, and in only one was the condition confined to the left kidney. In this case the patient had tuberculosis in the right lung. Where both kidneys were felt the right was more movable in all but three cases, the left not being the more movable in any.

Dr. Larrabee classifies the cases as follows: (1) palpable kidney, where the kidney can be felt only in deep inspiration; (2) movable kidney, where the organ can be held down during expiration; (3) floating kidney, where the organ can be pushed freely about. Thirty-nine of his cases belonged to the first class, 49 to the second, and 24 to the third. A study of the patients' ages made it appear that the occurrence most usually supervenes in young adult life. Marriage and pregnancy did not seem important factors, for movable kidney was found in 41 per cent. of the single women examined and in 44 per cent. of the married or widowed. More significance is probably due to emaciation, as 66 per cent. of those in whom movable kidney was found said they had lost weight. The condition was very much more commonly found in thin people than in others. Efforts to classify the cases according to the kinds of corsets worn or to determine the effects of tight lacing were unsuccessful.

Two patients of this series had been previously operated on. In one a nephrorraphy had been done, followed, as the organ was not retained in position, by a nephrectomy. The other kidney was movable, and the symptoms had not been relieved by the operation. In the other patient a sinus had remained for a year after the nephrorraphy, and the symptoms were not relieved. It would appear, therefore, that operation should be advised only as a last resort, and the uncertainty of its affording relief explained to the patient.

[1] Boston City Hospital Medical and Surgical Reports, 1903.

